# Immune-related adverse event-myocarditis with marked ST-segment elevation requiring differentiation from COVID-19-induced myocarditis: a case report

**DOI:** 10.1093/ehjcr/ytae370

**Published:** 2024-07-24

**Authors:** Kana Fujita, Yoshitaka Ohashi, Yoshinori Nagasawa, Tomoyuki Otani, Kinta Hatakeyama

**Affiliations:** Department of Cardiology, Ako City Hospital, 1090, Nakahiro, Ako, Hyogo 678-0232, Japan; Department of Cardiology, Ako City Hospital, 1090, Nakahiro, Ako, Hyogo 678-0232, Japan; Department of Cardiology, Konan Medical Center, 1-5-16, Kamokogahara, Higashinada-ku, Kobe 658-0064, Japan; Division of Pathology, Ako City Hospital, 1090, Nakahiro, Ako, Hyogo 678-0232, Japan; Department of Pathology, National Cerebral and Cardiovascular Center, 6-1 Kishibe-Shimmachi, Suita, Osaka 564-8565, Japan; Department of Pathology, National Cerebral and Cardiovascular Center, 6-1 Kishibe-Shimmachi, Suita, Osaka 564-8565, Japan

**Keywords:** Immune checkpoint inhibitors, Immune-related adverse events, Myocarditis, SARS-COV-2, Case report

## Abstract

**Background:**

Immunotherapy with immune checkpoint inhibitors (ICIs) enhances the host immune reaction against tumour cells by inhibiting intrinsic down-regulators of the T cell-mediated immune response. Although the advent of ICIs has dramatically changed oncology, ICIs may also trigger an overactivation of T cells against non-cancerous tissues, leading to off-target immune-related adverse events (irAEs).

**Case summary:**

A 64-year-old man with a history of seven courses of atezolizumab, an ICI, for small-cell lung cancer and coronavirus disease 2019 (COVID-19) was admitted to the hospital complaining of acute chest pain. Transthoracic echocardiography showed preserved ejection fraction (EF), but electrocardiography indicated precordial ST-elevations and marked increases in biomarkers for myocardial injury were observed. Emergent cardiac catheterization showed no significant coronary stenosis. On the fifth hospital day, EF decreased to 25% and pericardial effusion occurred. Endomyocardial biopsy was immediately performed, and prednisolone (60 mg/day) was administered. Troponin I level rapidly reduced, ST changed, and EF improved. Histological examinations demonstrated CD8-predominant T lymphocytic infiltration with myocardial cell injury, consistent with irAE-myocarditis.

**Discussion:**

In irAEs, myocarditis is the most common and severe cardiac manifestation with a high mortality. Even at 20 weeks after the initial ICI treatment, irAE-myocarditis occurs and the clinical presentation may mimic ST-elevation myocardial infarction. The histopathological findings suggested the high possibility of irAE-myocarditis rather than COVID-19-induced myocarditis, but COVID-19 has possibly played a role in the development of late-onset irAE-myocarditis. This educational case implies the importance of immediate recognition of irAE even after stable ICI treatment.

**Learning points**

- Compared to coronavirus disease 2019 (COVID-19)-induced myocarditis, which characteristically demonstrates macrophage-predominant infiltrates with a small number of T lymphocytes, immune-related adverse event (irAE)-myocarditis typically shows infiltration of predominantly CD8^+^ T cells on endomyocardial biopsy.
- When the diagnosis of irAE-myocarditis is suspected, interruption of immune checkpoint inhibitor (ICI) and urgent high-dose methylprednisolone treatment with endomyocardial biopsy should be considered.
- At even 20 weeks after initial ICI treatment, irAE-myocarditis occurs and it may mimic ST-elevation myocardial infarction.

## Introduction

Immunotherapy with immune checkpoint inhibitors (ICIs) enhances the host immune reaction against tumour cells by inhibiting the intrinsic down-regulation of the T cell-mediated immune response. Immune checkpoint inhibitors may also trigger an overactivation of T cells against non-cancerous tissues, leading to off-target immune-related adverse events (irAEs).^1^ During coronavirus disease 2019 (COVID-19), it was observed to elicit myocarditis, vasculitis, and thrombotic complications. Our case of late-onset cardiac manifestation of irAE after the infection demonstrates the emergent measures to the presentation mimicking acute coronary syndrome and the importance of endomyocardial biopsy and treatments with corticosteroids; it also provides insights into the mechanisms of irAE-myocarditis, overlapping with COVID-19.

## Summary figure

**Figure:**
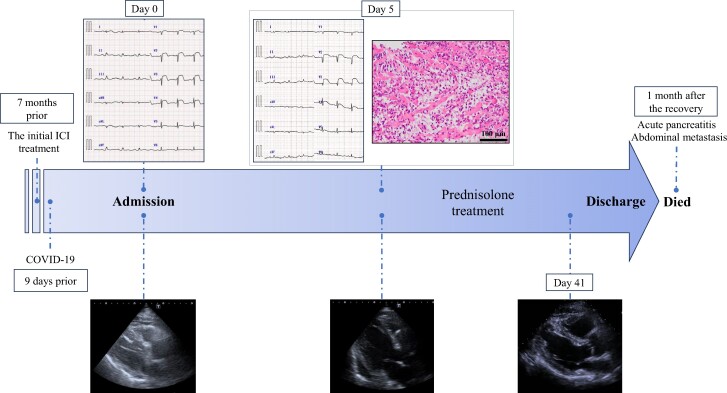


## Case presentation

A 64-year-old Japanese man with advanced small-cell lung cancer presented to the emergency department with a 2-day history of pleuritic chest pain and dyspnoea. Approximately 7 months prior, he received seven courses of atezolizumab, and his general condition was successfully stable. Nine days before the admission, he suffered from COVID-19.

The patient presented a temperature of 36.5°C, a relative high heart rate of 101 b.p.m., blood pressure of 119/68 mmHg, and an oxygen saturation of 95% when breathing ambient air. The heart rhythm was regular with no remarkable murmur. The lung sounds were clear with no rales. No oedema was observed in the extremities.

**Figure 1:**
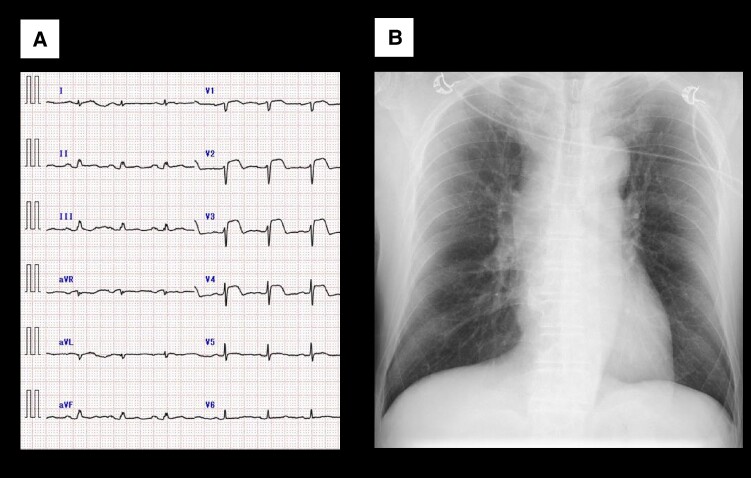
Electrocardiography on Day 0 of admission showing a normal sinus rhythm of 96 b.p.m. and marked precordial ST-segment elevations (*A*). Chest radiography does not show pleural effusion or any significant cardiomegaly with a cardiothoracic ratio of 44% (*B*).

Electrocardiography (ECG) showed marked precordial ST-segment elevation (*Figure 1A*). Initial blood sample showed elevated values of C-reactive protein (28.5 mg/L), creatine kinase (163 U/L), creatine kinase–MB (24 IU/L), NT-pro brain natriuretic peptide concentration (1422 pg/mL), and troponin I (TnI) (over 25 000 pg/mL), but normal values of haemoglobin (120 g/L) and white blood cells (7300/μL). Chest radiography did not show pleural effusion or significant cardiomegaly with a cardiothoracic ratio of 44% (*Figure 1B*). Transthoracic echocardiography (TTE) revealed that left ventricular (LV) contraction was preserved without ventricular dilatation (see Supplementary material online, *Video S1A*). Emergent coronary angiography was performed, demonstrating no significant coronary artery lesion; left ventriculography revealed that LV contraction was almost preserved with a global ejection fraction (EF) of 66%. After admission, TnI levels remained above the upper limit, and the ST-segment elevation was exacerbated and prolonged with elevations of other myocardial enzymes. Frequent atrial premature contraction and paroxysmal atrial fibrillation were observed, and systolic blood pressure was decreased below 70 mmHg. On Day 5, EF was decreased to 24% with diffuse wall motion abnormalities; pericardial effusion was observed (see Supplementary material online, *Video S1B*).

**Figure 2:**
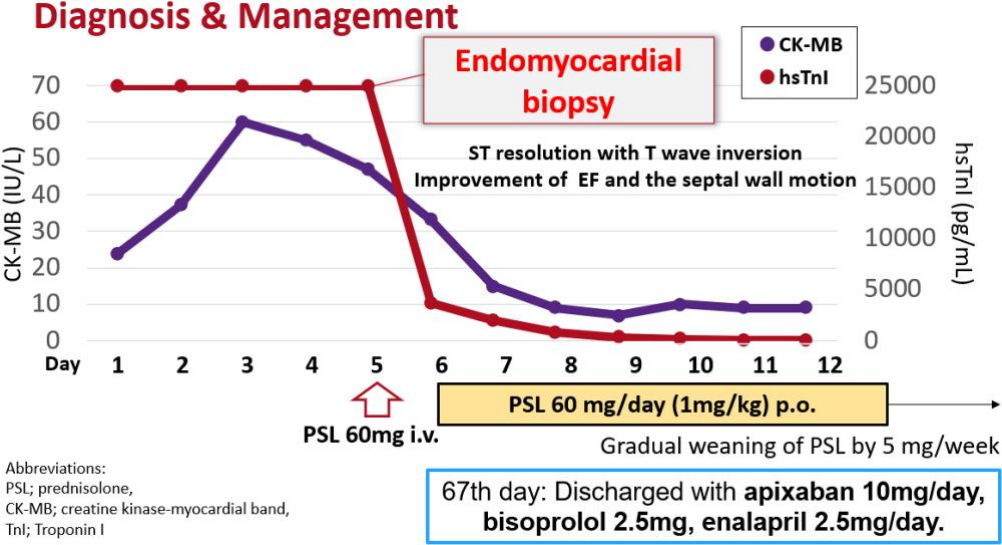
Clinical course of the case.

According to the 2017 European Society of Cardiology guidelines for the management of acute myocardial infarction with ST-segment elevation, emergent coronary angiography was performed for thrombotic tendency with COVID-19, but we could not find a culprit lesion corresponding to the regional ST change. Since the results of all myocarditis-causing viral tests were negative, myocarditis due to irAE or the SARS-COV-2 virus was suspected. Therefore, endomyocardial biopsy was performed and the treatment with intravenous prednisolone was started (*Figure 2*). Because TnI rapidly decreased on the Day 6, steroid treatment was switched to oral administration. During a gradual weaning of prednisolone, an ST resolution with T-wave inversion was indicated, EF was improved to 50%, and the septal wall motion also improved (see Supplementary material online, *Video S1C*). On Day 67, the patient was finally discharged.

**Figure 3:**
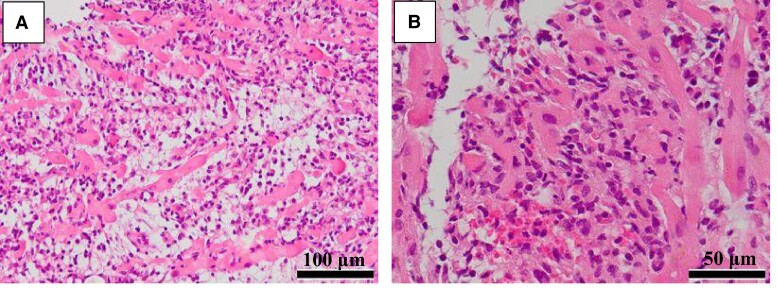
Cardiomyocyte injury is evident with the disruption and tapering of myocardial cells (*A*). There is accompanying interstitial oedema (*A*) and haemorrhage (*B*). (*A*) Scale bar = 100 μm; original magnification, ×200. (*B*) Scale bar = 50 μm; original, magnification, ×400.

**Figure 4:**
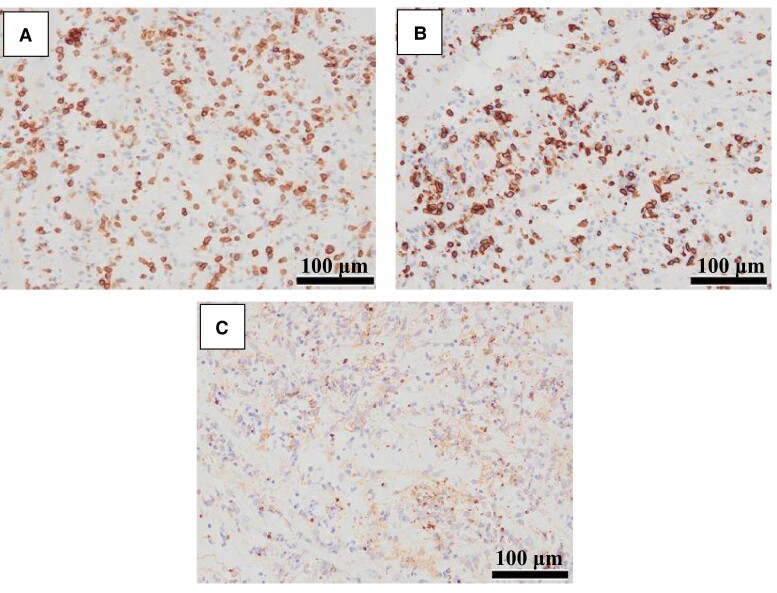
Immunohistochemistry revealing infiltrating inflammatory cells consisting predominantly of CD3-positive T lymphocytes (*A*); most of which are CD8 positive (*B*). Some CD68-positive macrophages are admixed (*C*). Scale bar = 100 μm; original magnification, ×200.

Right ventricular endomyocardial biopsy demonstrated dense inflammatory cell infiltration with myocyte injury, which was evidenced by the disruption and tapering of myocardial cells (*Figure 3A*). There was also interstitial oedema and haemorrhage (*Figure 3B*). Immunostaining showed that inflammatory infiltrate consisted predominantly of CD3-positive T lymphocytes (*Figure 4A*), most of which were CD8 positive (*Figure 4B*). Some CD68-positive macrophages were admixed (*Figure 4C*). These findings were consistent with those reported in irAE-myocarditis. In contrast, endomyocardial biopsies from patients with COVID-19 show macrophage-predominant infiltrates with a small number of T lymphocytes. The diagnosis of irAE-myocarditis was made. Thus, the interruption of ICI was inevitable.

One month after the recovery of myocarditis, the patient unfortunately developed acute pancreatitis with an abdominal metastasis and then died, possibly due to the interruption of ICI.

## Discussion

Since the award of the Nobel Prize to Tasuku Honjo, cancer immunotherapy using ICIs has shown efficacy in a variety of malignancies. By blocking the checkpoint molecules such as CTLA-4, PD-1, and PD-L1 from binding with their partner proteins, ICIs inhibit the ‘off’ signal, activating T cells and promoting the killing of cancer cells. Since PD-L1 is expressed in a variety of cells including non-cancerous tissues, the use of ICIs can lead to an inappropriate autoimmune response, resulting in irAEs.

Myocarditis is the most common and severe cardiac manifestation with a high mortality rate in irAE. It frequently develops during the first 12 weeks of initial ICI treatment, although late-onset cases may occur after 20 weeks.^1^ In irAE-myocarditis, an abnormal ECG and troponin elevation occur earlier than echocardiographic and biochemical findings of cardiac dysfunction.^2^ It may not always present new conduction blocks, decreased voltage, and repolarization abnormalities, but also shows ST-elevation with preserved systolic function.

Endomyocardial biopsy is the gold standard diagnostic procedure, and the immunohistochemical staining of irAE-myocarditis typically shows predominantly CD8^+^ T cells interspersed with CD4^+^ T cells and macrophages.^3^ In addition, several articles^3,4^ have described the up-regulation and positive staining of PD-L1 in myocardial tissue in irAE-myocarditis. Interruption of ICI is recommended in all cases of suspected irAE-myocarditis while investigations are performed.^5^ Once the diagnosis is considered likely, treatment of irAE-myocarditis with steroids should be started immediately.

Late-onset cases may occur because PD-1 and PD-L1 remain occupied for >1 year after the last infusion of the ICI.^6^ One cohort study indicated that heart failure with LV systolic dysfunction was more common in late-onset cases; thus, there may be a possible mechanism for sustained myocardial damage by the dysregulation of cardiomyocyte Ca^2+^ currents.^7^ We reported a unique case with late-onset cardiac manifestations of irAE immediately after the SARS-CoV-2 infection, despite stable ICI treatments for 7 months; it led us to consider that the SARS-CoV-2 infection might act as a trigger. Several reports, including case series^8^ and case reports,^9,10^ have advocated that the ICIs and COVID-19 could simultaneously promote adverse immune hyperactivation, potentially resulting in irAEs. Cytokine release syndrome by ICIs is a rare phenomenon; however, it places patients at an elevated risk of mortality. COVID-19 also presents a cytokine storm. Recently, persistent stimulation by the virus was reported to induce reduced T cell function and a loss of T cell-related cytokine production, shifting from a status of hyperactivation to exhaustion. This increases levels of PD-1, leading dysregulation in several types of immune cells.^11^ Enhanced PD-L1 expression could be a cardioprotective mechanism against myocardial injury^12^; it provides infected cells with an immune escape to both innate and adaptive immune responses. In fact, the overactivation of T cells tends to contribute to severe immune injury under the blockade of PD-L1. However, the hypothesis of irAE-myocarditis due to a combination between ICI and COVID-19 mechanism must still be proved and steps must be taken to prove it.

COVID-19-associated myocarditis alone is mainly transient and less severe than accompanying irAE-myocarditis. In general, permanent discontinuation of ICI treatments is recommended with grade 4 toxicities.^13^ However, the patient passed away relatively early after the remission of myocarditis. It is important to hold multidisciplinary team discussions to balance the risk/beneﬁt of cancer treatment discontinuation when patients with cancer are infected with SARS-COV-2.

## Supplementary Material

ytae370_Supplementary_Data

## Data Availability

The data underlying this article are available in the article and in its online Supplementary material.

## Lead author biography

After graduating from Okayama University in 2020, the author traversed the path of general internal medicine at Ako City Hospital after 2 years of residency at the Hyogo Prefectural Tamba Medical Center.

## Notes

**Consent:** The authors confirm that written informed consent for submission and publication of this case report, including images and associated text, has been obtained from the patient’s next of kin in line with COPE guidance.

**Funding:** None declared.
